# Selected abstracts from the 25th Annual Meeting of the Society in Europe for Simulation Applied to Medicine: Glasgow, United Kingdom. 12-14 June 2019

**DOI:** 10.1186/s41077-019-0104-y

**Published:** 2019-06-26

Publication of this supplement was funded by the Society. The Supplement Editors are all members of the Advances in Simulation editorial board, and they declare no competing interests.

Edited by Tanja Manser, Dinker Pai, Gabriel Reedy and Cathy Smith

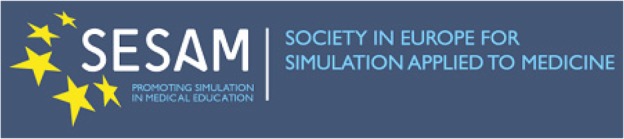



## A1 Can simulation based training for emergency medical teams improve patient safety? A systematic literature review

### Kjetil Torgeirsen

#### SAFER, Stavanger, Norway

**Correspondence:** Kjetil Torgeirsen (kjetil.torgeirsen@dabb.no)

**Introduction & Aims**

Health care is often organized as inter-professional team-work. Most health care professionals have little or no knowledge or training in how to get teams to work from their education. Most of the nontechnical skills are rarely practiced in isolation by any health care professional even though it is pointed out that nontechnical skills can mitigate risk of errors or adverse events. Characteristics of emergency medical teamwork is; complex, uncontrolled situations/scenes, high levels of stress and high stakes. These factors can individually increase the risk of errors and adverse events and when present together the risk increases. It is important to use highly efficient tools to reduce this risk. Research question: Can simulation based training for emergency medical teams improve outcomes and patient safety?

**Methods**

A systematic literature review was conducted based on a PICO analysis with assistance from a librarian searching Medline and Embase. Inclusion criteria for the abstracts were based on a PICO analysis; Population; clinicians in some kind of emergency situation and multidisciplinary teams. Educational articles with student populations were excluded as they are not likely to measure patient outcome. Intervention – simulation based training. Outcome/endpoints: impact on patient outcome (reduced risk of errors or reduced mortality) Kirkpatrick level 4 (K4).

**Results & Discussion**

Of the initial 164 studies, 18 studies met the inclusion criteria. 6 of the included studies were systematic literature reviews conducted in the period 2010 - 2014. 10 single studies found impact on K4 level and mortality. There seems to be an increasing number of studies proving reduced mortality after simulation based training intervention. Previous literature reviews do not include new studies, and have missed important studies. The findings from this literature search indicate a potential huge impact on patient outcome with several studies reporting of 18 – 50% reduced mortality rates and also for other indicators for patient safety. The other main finding in this review article is the lack of reporting guidelines for medical simulation studies and the difficulties of getting an overview over the research in this field. None of the included studies involve pre-hospital health care providers or teams, but medical emergencies and interdisciplinary teams.

**Ethics Statement**

This study did not require formal ethics approval because it was a literature review.

## A2 Comparison of two mask holding techniques for one-person bag-mask ventilation in mannequin studies

### Elīna Zvaigzne, Oļegs Sabeļņikovs, Ruta Jakušonoka

#### Rīga Stradiņš University, Rīga, Latvia

**Correspondence:** Oļegs Sabeļņikovs (olegs.sabelnikovs@rsu.lv)

**Introduction & Aims**

Effectively performed bag-mask ventilation remains an essential component of cardiopulmonary resuscitation (CPR). Correct mask holding technique ensures open airways by optimal head-neck alignment and tight seal between face and mask. Despite it being seemingly simple manipulation, standard mask holding method (“EC”clamp”) might prove to be challenging, especially in situation when only one rescuer is presented. Alternative may be “glass-holding” technic previously described for bag-mask ventilation in children. Study hypothesis is that “glass-holding” clamp could be relatively easiest technique (comparing with “EC clamp”) for holding mask in adult patients in case of one rescuer CPR when only the side access to a patient is available. The aim of the study was to compare a tidal volume provided with both methods. During adult cardiopulmonary resuscitation tidal volumes of approximately 500 to 600 mL (6 to 7 mL/kg) should suffice and each rescue breath provide over one second regarding to European Resuscitation Council Guidelines for Resuscitation 2015.

**Methods**

Research took place in Medical Education Technology Center of the Riga Stradins University in October 2018. Thirty-nine medical students performed a bag-mask ventilation on Mannequin “ResusciAnne QCPR” (Laerdal Medical). Each one provided 5 ventilations using “EC” clamp, followed by 5 ventilations using “glass-holding” clamp sitting aside from patient. Tidal volume of ventilations provided was registered with Skill reporter software (Laerdal Medical). Median volume of ventilations provided by each subject using each clamp were compared.

**Results & Discussion**

Totally 390 ventilations were measured. The tidal volume measurements show that the “EC” clamp yielded a median of 594 (IQR 527-630.8) ml. The “glass-holding” clamp yielded a median of 611m (IQR 569.4-634.4) ml (z=2.875, p=0.004). Using “EC“ method, tidal volume >500 ml was reached fewer times than using alternative method (cumulative percentage 12.8% and 5.1%, respectively). Results of the study shows possibility to reach a recommended tidal volume using “glass-holding” clamp.

**Ethics Statement**

The authors declare that they have followed the guidelines for scientific integrity and professional ethics. The article does not contain any studies with human or animal subjects.

## A3 Development of a practical feedback tool for trauma leadership

### Nico Leenstra^1^, Oliver Jung^2^, Jaap Tulleken^3^

#### ^1^Wenckebach Institute, University of Groningen, University Medical Center Groningen, Groningen, the Netherlands; ^2^Department of Anesthesiology, University of Groningen, University Medical Center Groningen, Groningen, the Netherlands; ^3^Department of Critical Care, University of Groningen, University Medical Center Groningen, Groningen, the Netherlands

**Correspondence:** Nico Leenstra (n.leenstra@hotmail.com)

**Introduction & Aims**

Trauma leadership training is increasingly being incorporated into trauma courses. An increasing demand is thereby put on educators’ ability to assess, reflect on, and improve leaders’ nontechnical skills. However, there is a paucity of tools that can support educators’ targeted observation and feedback. We therefore developed a novel feedback tool that focuses specifically on the trauma leader. We based this tool on previous work, in which our task analyses of trauma leadership resulted in a granular skill taxonomy, called the “Taxonomy of Trauma Leadership Skills” (TTLS). The central aims in the present study were to enhance the TTLS’ practicality for in-action assessments and notetaking, and to improve its elements for being sufficiently instructive as ‘stand-alone’ items to guide observations and the identification of learning points.

**Methods**

We subjected the TTLS to practical evaluation and modification in two stages. In the first stage – consisting of three rounds – testing panels reviewed and improved the elements’ clarity, observability and brevity. They were asked to observe brief video vignettes from the local trauma team training and indicate from the list of elements which behavior or behaviors they felt were being shown. Any ambiguity, redundancy or difficulties in identifying behaviors objectively were addressed by re-phrasing, combining or omitting elements. This resulted in a prototype feedback sheet. The second stage consisted of a round of testing and a final evaluation in actual practice. In the ATLS refresher course in the Netherlands, ATLS instructors (6 and 16 respectively) used prototype feedback sheets to collect impressions on the trauma leaders’ performances and to debrief the scenario. Afterwards, they filled out a questionnaire regarding the sheet’s clarity, content validity, ease of use, usefulness and impact on instructor tasks. Answers were given on a 3-point scale (no-moderately-yes).

**Results & Discussion**

The final feedback tool, called the TTLS for Assessment (TTLS-A), contains 5 skill categories (information coordination, action coordination, decision making, communication, and coaching and team development) and an additional skill set for the briefing phase of trauma care. The TTLS-A was evaluated as being complete, clear, practical and helpful at multiple stages of the training. In relation to previous trauma assessment tools, the TTLS-A provides an additional level of specificity that is pivotal for detailed recommendations for targeted practice. The positive evaluations, by trauma instructors from across the Netherlands, suggest that the TTLS-A is a valid and helpful tool for training trauma team leaders’ nontechnical skills.

**Ethics statement**

The authors declare that they have followed the guidelines for scientific integrity and professional ethics. The article does not contain any studies with human or animal subjects.

## A4 Evaluating pilot data from a national simulated programme in Scotland focusing on challenging communication scenarios for junior doctors

### Chris O'Shea, Jennie Higgs, Nathan Oliver, Joe Roberts

#### NHS Lothian, Scotland, UK

**Correspondence:** Chris O'Shea (chris.o'shea@nhs.net)

**Introduction & Aims**

Junior doctors in the UK face a significant increase in responsibility when transitioning from their first to second year (known as Foundation Year 2, or FY2). Research by NHS Lothian found FY2 doctors feel ill-prepared for this, especially in managing difficult conversations. They struggle to evidence communication skills competencies in appraisals. NHS Education for Scotland wished to address this by adopting a national simulation programme focusing on areas of challenging communication. The aim is for all FY2 doctors in Scotland to receive this training. Such interventions are resource intensive. Effective evaluation is vital. Over 2017-2018 all FY2 doctors in South-East Scotland had the opportunity to attend the training.

We were asked to pilot an evaluation to inform the national approach. Our aims were to assess the impact of attending the course on perceived trainee confidence and to demonstrate if this method of assessment could be adopted nationally.

**Methods**

Over 2017-2018, 171 out of 199 FY2 doctors in South-East Scotland attended training encompassing four challenging communication scenarios: assertiveness under pressure, recognising vulnerable adults, dealing with confrontation and anger, and discussing end of life care. The session design is informed by nationally agreed learning objectives aligned to the Foundation Programme.

All participants were invited to complete a pre-course and post course questionnaire. Follow up questionnaires were sent via email 3 months after attendance. These consisted of Likert scales rating confidence in addressing challenging communication situations related to the scenarios.

**Results & Discussion**

All attendees gave informed consent to participate in the evaluation. We collected 171 pre-course questionnaires, 171 post course questionnaires, and 62 3 monthly responses (a 36% response rate).

Statistically significant increases in attendee confidence were observed in managing all scenarios immediately following the course (Table 1). This confidence was sustained when surveyed three months later. There was a statistically significant increase in confidence in managing challenging mental health scenarios from immediately after attending the course to three months later.

We have demonstrated FY2 doctors had significantly increased confidence in their ability to tackle challenging communication situations aligned to their curriculum following attendance at the course. Importantly this improvement was not lost 3 months later. We have informed the national approach by showing a 3-month email survey can yield acceptable response rates. One limitation in our approach was a lack of control data. Identifying this at the pilot stage led to the national questionnaires incorporating appropriate statistical process controls.

**Ethics Statement**

The authors declare that all procedures followed were in accordance with the ethical standards of the responsible committee on human experimentation (institutional and national) and with the Helsinki Declaration of 1975 (In its most recently amended version). Informed consent was obtained from all patients/participants included in the study. All institutional and national guidelines for the care and use of laboratory animals were followed.

**Table 1 (abstract A4).** Two-Sample T-Test Comparison of participants mean confidence pre, post, and 3 months after the training. Confidence was rated by 5 point Likert Scale (1 = not at all confident, 5 = very confident)

	Discussions around end of life care	Being assertive under pressure	Dealing with confrontation/anger in the workplace	Dealing with difficult mental health scenarios
Confidence Totals				
Pre-Course Mean (SD), n=171	3.3 (0.8)	2.8 (0.7)	2.8 (0.8)	2.6 (0.8)
Post-Course Mean (SD), n=171	4.1 (0.5)	3.8 (0.6)	3.8 (0.6)	3.5 (0.7)
3 Month Mean (SD), n = 62	3.9 (0.6)	3.6 (0.8)	3.7 (0.7)	3.8 (0.7
Two-Sample T-Test Comparisons *(p)*				
Pre-course → post course	<0.001	<0.001	<0.001	<0.001
Pre-course → 3 month	<0.001	<0.001	<0.001	<0.001
Post course →3 month	0.30	0.97	0.25	0.02

## A5 Evaluation of nontechnical skills of novice nursing and medical students at interprofessional simulation setting

### Senay Sarmasoglu, Cigdem Yucel, Gulten Koc, Melih Elcin

#### Hacettepe University, Ankara, Turkey

**Correspondence:**  Senay  Sarmasoglu  (senay.sarmasoglu@hacettepe.edu.tr)

**Introduction & Aims**

Nontechnical skills (NTS) are the cognitive and interpersonal skills, supplementing clinical and technical skills, necessary to ensure safe patient care. The purpose of the study is exploring the NTS of novice nursing and medical students at interprofessional simulation setting.

**Methods**

The study has been conducted with novice nursing and medical students who participated in interprofessional simulation within the context of interprofessional course in 2018-2019 Fall Semester. As an interprofessional team one nursing and one medicine student interacted with a standardized patient. Two researchers watched the videos of simulated cases simultaneously and assessed the performance of interprofessional student teams by using the Anaesthesiologists' Non-Technical Skills in Denmark (ANTSdk) Rating Form. Since the interprofessional course will be continued until the end of the semester, the rating the videos is still ongoing process, so abstract includes preliminary data and results of randomly selected eighteen videos from first three weeks of the course.

**Results & Discussion**

According to the preliminary data, 55.5 % of interprofessional teams had higher scores on situational awareness category, 28.0 % of teams had higher scores on leadership category 11.0 % of teams had higher scores on teamwork and 5.5 % had higher scores on decision making category. The results of this study might raise students’ awareness related with the importance of non-technical skills and their contribution to patient care. Moreover, the results of this study might help educators to get understanding about students’ basal nontechnical skills performance in order to promote learning facilities.

**Ethics statement**

The authors declare that all procedures followed were in accordance with the ethical standards of the responsible committee on human experimentation (institutional and national) and with the Helsinki Declaration of 1975 (In its most recently amended version). Informed consent was obtained from all patients/participants included in the study.

## A6 Evaluation of novel chest compression method with the use of a high fidelity neonatal simulator

### Jacek Smereka^1^, Michal Pruc^2^, Kurt Ruetzler^3^, Lukasz Szarpak^4^

#### ^1^Department of Emergency Medical Service, Wroclaw Medical University, Wroclaw, Poland; ^2^Lazarski University Zuzanna Popielarska Lazarski University Klaudia Kulak Lazarski University Dominika Dunder Lazarski University, Poland; ^3^Departments of General Anesthesiology and Outcomes Research, Cleveland Clinic, Cleveland, USA; ^4^Lazarski University, Warszawa, Poland

**Correspondence:** Lukasz Szarpak (lukasz.szarpak@gmail.com)

**Introduction & Aims**

The ability to perform high quality chest compressions is one of the basic skills that should be performed by medical personnel, especially paramedics. The European Resuscitation Council guidelines on neonatal resuscitation indicate that chest compression should be carried out using one of two techniques: two fingers (TFT), consisting of two fingers of one hand perpendicular to the chest; two thumbs (TTHT), where the other fingers encircling the patient's chest and provide support for the chest. However, both techniques have their advantages and disadvantages. The aim of the study was to evaluate a new chest compression technique developed by Smereka & Szarpak during newborn resuscitation performed by medical students.

**Methods**

The study involved 45 medical students who were not yet trained in infant or neonatal resuscitation, and have not performed any clinical or simulation resuscitation. Prior to the study, they participated in a 2-hour training in neonatal resuscitation, including physiology, neonatal pathophysiology, as well as a discussion and instruction on chest compression techniques recommended by the ERC. In addition, a third technique of chest compression, consisting of two thumbs perpendicular to the chest to form an extension of the forearms (nTTT; Figure 1) developed by Smereka & Szarpak, was demonstrated to the participants. Then participants took part in a 30-minute practical training during which, under the supervision of an instructor, they performed cardiopulmonary resuscitation with the use of three compression techniques. Tory® S2210 Tetherless and Wireless Full-term Neonatal Simulator (Gaumard Scientific, Miami, FL, USA) was used to simulate a newborn requiring resuscitation.

**Results & Discussion**

The chest compression rates for the studied chest compression techniques varied between 133 [IQR; 122-139] for TFT, 118 [IQR; 115-122] for TTHT, and 110 [IQR; 110-125] for nTTT. The median compression depth with TFT, TTHT and nTTT was 27, 42 and 41 mm, respectively. The degree of full chest recoil was 94% for TFT and nTTT and only 29% for TTHT. In all three techniques, the correctness of chest compressions ranged from 98 to 100%. The results show that the methods recommended by ERC (TFT and TTHT) have both advantages and disadvantages. The TFT compression technique, compared to TTHT, is worse for the depth of chest compressions, however, the chest relaxes much better. In the case of the evaluated nTTT technique, it is a combination of these techniques so that both the depth of compressions and the degree of chest recoil are optimal.

**Ethics statement**

The authors declare that all procedures followed were in accordance with the ethical standards of the responsible committee on human experimentation (institutional and national) and with the Helsinki Declaration of 1975 (In its most recently amended version). Informed consent was obtained from all patients/participants included in the study. All institutional and national guidelines for the care and use of laboratory animals were followed.

**Figure 1 (abstract A6).**
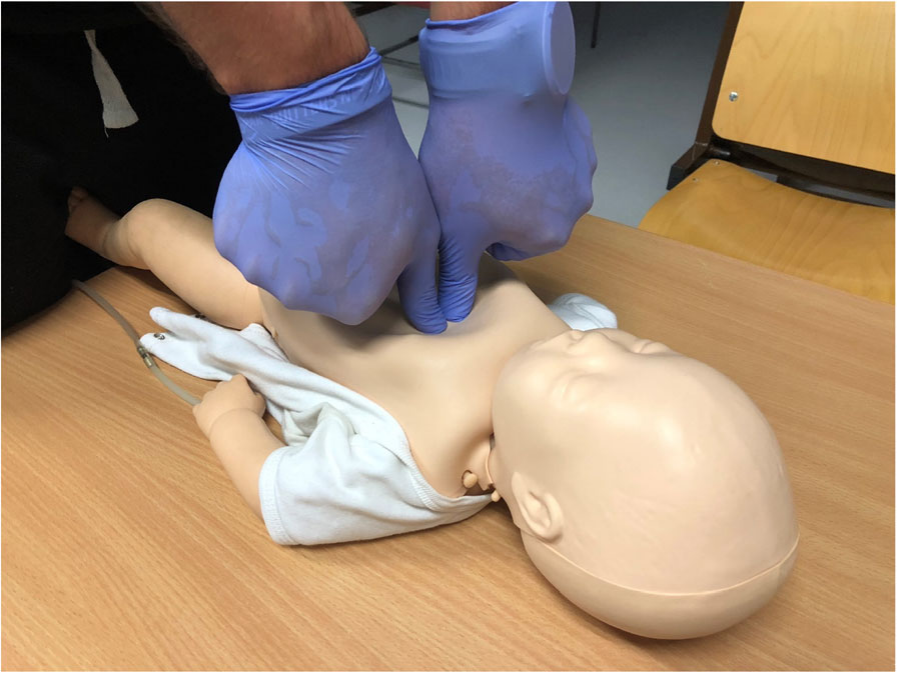
See text for description

## A7 Eye tracking to assess multiprofessional situation awareness of a simulated PPH management

### Arnaud Desvergez^1^, Malik Boukerrou^2^, Jean-Bernard Gouyon^3^, Arnaud Winer^1^, Médéric Descoins^4^

#### ^1^Critical care unit, Centre de Simulation en Santé de l'Océan Indien, Centre d'Etudes Périnatales de l'Océan Indien (EA 7388), CHU Saint-Pierre de la Réunion, Université de la Réunion; ^2^Gynecolog unit, Centre de Simulation en Santé de l'Océan Indien, Centre d'Etudes Périnatales de l'Océan Indien (EA 7388), CHU Saint-Pierre de la Réunion, Université de la Réunion; ^3^Centre d'Etudes Périnatales de l'Océan Indien (EA 7388), CHU Saint-Pierre de la Réunion, Université de la Réunion; ^4^Centre de Simulation en Santé de l'Océan Indien, Centre d'Etudes Périnatales de l'Océan Indien (EA 7388), CHU Saint-Pierre de la Réunion

**Correspondence:** Arnaud Desvergez (desvergez.a@gmail.com)

**Introduction & Aims**

The management of postpartum hemorrhage (PPH) is an emergency requiring the intervention of several professionals. The eyes are the reflection of the cognitive processes involved in emergency situation. Visual behavior is an aggregate of learning reinforced by profession and experience. It is the support of Situational Awareness (SA) which is a cognitive system that links the physician’s visual perception to his mental representation of a situation. We dissociate the SA into 3 levels (SA level 1: perception, SA level 2: comprehension and SA level 3: anticipation). The aim of this study is to compare the visual behavior and the related level of SA of physicians from two different specialties—Anesthesiologists (AR) and Obstetricians (GO)—faced with the same situation of simulated PPH.

**Methods**

The visual behavior of AR and GO was analyzed by an eye-tracking device when viewing a simulated PPH video. The scenario was a severe PPH complicated by a state of hemorrhagic shock. Heat maps were extracted at different times during the management. Regions of interest (ROI) were previously defined on the video. Associated number of fixation (NBF) and the fixation duration mean (FDM) were measured and compared between groups by Wilcoxon test. SAGAT (Situation Awareness Global Assessment Technique) questionnaires were administered during the viewing to compare the level of SA between our groups by ANOVA. The significance is retained at 5%.

**Results & Discussion**

30 AR and 32 G0 were included. Heat maps highlight a different distribution of visual attention between specialties (Figure 1). Statistically, GOs looked more often and longer the ROIs corresponding to the perineal area, blood loss, obstetricians and his equipment. While MARs looked more closely at the patient’s vital parameters (heart rate, blood pressure, respiratory rate and ECG pattern) The level of SA is significantly higher in AR group (67 ± 7% vs. 60 ± 11% in GO, p <0.05). Levels 1 (Perception) and 3 (Anticipation) are significantly higher in AR than in GO group (respectively SA 1: 71 ±11% vs. 64 ± 13% and SA 3: 72 ±10% vs. 61 ± 16%, p <0.05).

ARs and GOs engaged in the same clinical situation develop different behavior and visual attention. These differences in integrated perception reflect a different cognitive functioning resulting in a difference in SA. We need to optimize our professional communication to facilitate the transfer of relevant information between professionals and to construct a group SA.

**Ethics statement**

The authors declare that all procedures followed were in accordance with the ethical standards of the responsible committee on human experimentation (institutional and national ) and with the Helsinki Declaration of 1975**.**

**Figure 1 (abstract A7).**
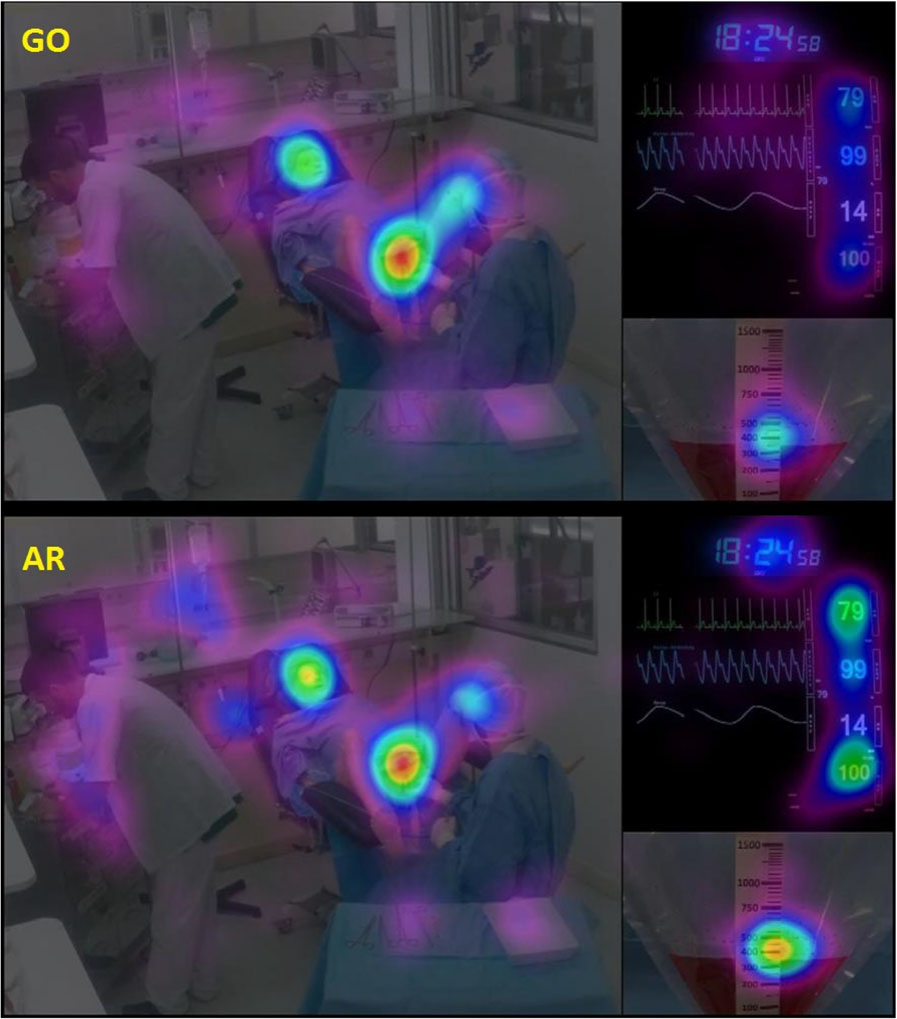
See text for description

## A8 From rehearsal to reality – how are junior doctors using their simulated experiences in their future practice?

### Jennie Higgs, Chris O'Shea, Nathan Oliver, Joe Roberts

#### NHS Lothian, Scotland, UK

**Correspondence:** Chris O'Shea (chris.o'shea@nhs.net)

**Introduction & Aims**

Junior doctors in their second year of training in Scotland (known as FY2 doctors) now take part in a national simulation programme. This programme focuses on challenging communication scenarios aligned to their curriculum. The Foundation Programme curriculum closely mirrors the principles of Scotland’s Chief Medical Officer’s vision of Realistic Medicine. However, local research has found FY2 doctors do not feel confident or comfortable in discussing core parts of the Realistic Medicine agenda with patients and colleagues.

South-East Scotland served as the pilot centre for evaluating the national FY2 simulation programme in 2017-2018. Our aims were to gather information regarding whether participants felt they were utilising the learning from the course in their day-to-day work. Participants in simulated activities often only provide feedback immediately following a course. We introduced follow-up questionnaires 3 months after they attended to capture self-reported data on the attendees’ perceived impact of the course on their practice.

**Description**

Over 2017-2018, 171 FY2 doctors rotating in South-East Scotland attended a half-day session comprising of four scenarios: being assertive under pressure, recognising vulnerable adults, dealing with anger and complaints, and discussing end of life care. With informed consent, we emailed participants three months after the course. We invited them to complete a survey which included free-text responses specifically asking for examples where they had used the course in their practice. We then asked participants to appraise how this went, and whether they would have done something differently prior to attending the course.

**Discussion**

62 participants completed the three-monthly follow up survey. The majority provided concrete examples of when and how they had used learning from the course in their practice. The examples closely matched the learning objectives set out at the training. Participants felt more able to have conversations aligned to the principles of Realistic Medicine. Specifically, they felt better prepared to have conversations around shared decision making and personalised patient care. Thematic analysis identified participants as feeling timid or nervous when facing these scenarios previously. Participants most frequently applied the skills learned on the course to conversations around end of life care and resuscitation.

We are pleased FY2 doctors feel empowered to make care more patient centred as a result of attending the training. They were able to provide meaningful examples of taking their learning forward. Following the success of this pilot study, the 2018-2019 national evaluation has elected to adopt this approach.

**Ethics statement**

The authors declare that all procedures followed were in accordance with the ethical standards of the responsible committee on human experimentation (institutional and national) and with the Helsinki Declaration of 1975 ( In its most recently amended version ). Informed consent was obtained from all patients/participants included in the study. All institutional and national guidelines for the care and use of laboratory animals were followed.

## A9 How does a simulated ward round change beliefs on technical and non-technical skills: an interprofessional comparison

### Chris Schnieke-Kind, Jennie Higgs, Alison Mackie, Chris O'Shea

#### NHS Lothian, Scotland, UK

**Correspondence:** Chris O'Shea (chris.o'shea@nhs.net)

**Introduction & Aims**

Doctors, nurses and pharmacists must work together effectively to provide high quality care. There are few opportunities for interprofessional learning at undergraduate level. University of Edinburgh (UoE), in collaboration with NHS Lothian (NHSL), have developed an innovative interprofessional simulated ward round for UoE final year medical and nursing undergraduates and NHSL pre-registration hospital pharmacists. Our aim was to understand what these respective disciplines thought were the most important technical skills (TS) and non-technical skills (NTS) for ward round participation, before and after our intervention.

**Description**

We ran 45 high fidelity ward rounds with simulated patients followed by a 40 minute debrief. This was delivered to 199 medical students, 28 nursing students and 8 pre-registration pharmacists.

We asked all participants to rank what they had thought were the 3 most important TS and NTS for proficient ward round practice prior to the simulated ward round and what they now thought having completed it. TS were: dealing with sick patients, dealing with phone calls, inpatient prescription (IP) tasks, writing in patient notes, requesting investigations, developing a job list, discharge prescription tasks. NTS were: prioritisation, understanding of patient background, multitasking, making clinical decisions, dealing with hierarchies, listening to the patient, delegating tasks. For all professional groups rankings prior to and following the ward round were analysed.

**Discussion**

Medical students showed a shift in TS from ranking individual practical tasks, including writing notes and IPs, to developing a job list to co-ordinate/delegate tasks. A shift in NTS was also observed. Emphasis changed from single, specific tasks towards task management, prioritisation and delegation.

Nursing students showed no shift in choosing TS, though there was some increase in the ranking of the importance of maintaining a ‘jobs list’. There was a shift in NTS which was closely mirrored the medical students, away from single-task related skills towards those focusing on broader management.

Finally, pre-registration pharmacists displayed a shift away from ‘multitasking’ towards ‘prioritisation’, suggesting a greater focus on single tasks than the management of many.

These shifts may reflect participants being challenged to act up into their prospective roles. We suspect these changes reflect their realisation of the need to move towards a co-ordinated, collaborative approach.

Our data supports the position that learners from three separate professions can be taught together. Having the opportunity to rehearse skills in a safe and supported environment can lead to a real shift in understanding.

**Ethics statement**

The authors declare that all procedures followed were in accordance with the ethical standards of the responsible committee on human experimentation (institutional and national) and with the Helsinki Declaration of 1975 (In its most recently amended version). Informed consent was obtained from all patients/participants included in the study. All institutional and national guidelines for the care and use of laboratory animals were followed.

**Figure 1 (abstract A9).**
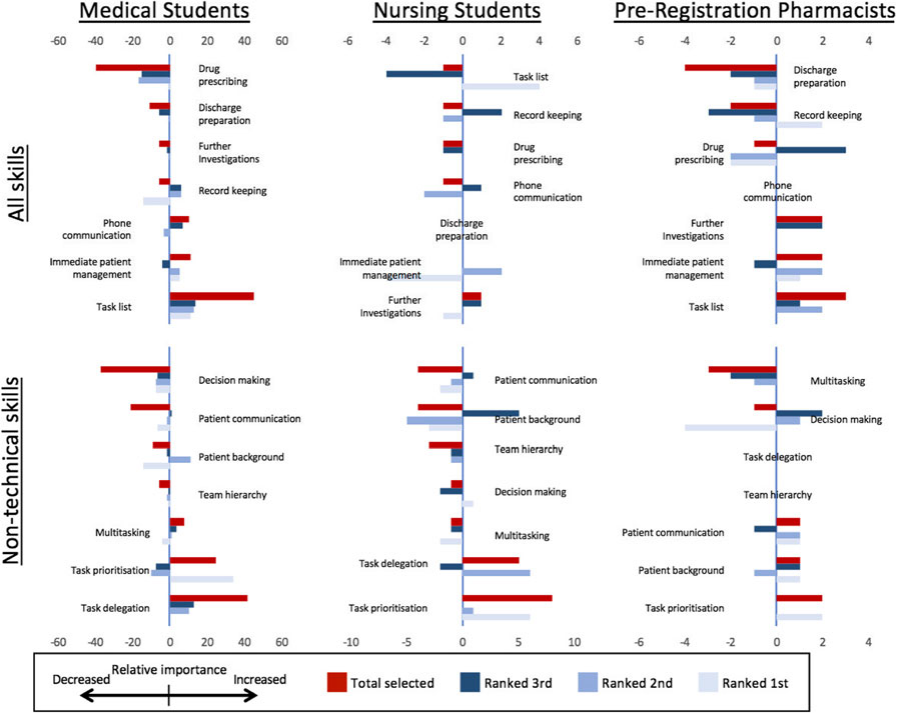
See text for description

## A10 Improving Bag-Valve-Mask (BVM) ventilation quality of 3rd year medical students using real-time simulation feedback in preparation for Entrustable Professional Activity (EPA) #12

### Vaia Abatzis^1^, Stephen Eason^2^, Keith Littlewood^1^

#### ^1^University of Virginia School of Medicine, Department of Anesthesiology, Charlottesville, USA; ^2^University of Virginia School of Medicine Lisa Morton University of Virginia School of Medicine, Department of Anesthesiology, Charlottesville, USA

**Correspondence:** Vaia Abatzis (vta4n@virginia.edu)

**Introduction & Aims**

BVM ventilation is critical to learn and master in medical school, however it is not always feasible to teach or evaluate BVM ventilation in real time with patients. The Association of American Medical Colleges (AAMC) now considers mastery of BVM ventilation imperative for medical school graduation as part of the EPAs. Simulation offers a unique, safe opportunity for medical students to improve their BVM ventilation technique as well as master (perform unsupervised) BVM ventilation as required for EPA #12.

The purpose of the research we present here is to investigate the ability to use simulation to objectively improve BVM ventilation in preparation for EPA (Entrustable Professional Activity) #12: perform general procedures of a physician. Our null hypothesis is there is no difference in BVM ventilation when comparing pre-treatment (Day 0) with post-treatment (Day 2) BVM ventilation data. Day 1 “treatment” is real-time BVM ventilation feedback from the mannequin.

**Methods**

3rd year medical students performed BVM ventilation three time using the Laerdal SimMan 3G during their Anesthesiology Peri-operative clinical clerkship. On Day 0 (baseline) they performed BVM ventilation for 2 minutes without feedback from the mannequin (“blinded”). On Day 1 they performed BVM ventilation while receiving real-time mannequin feedback displayed on a computer screen (rate, volume). On Day 2 they once again performed BVM ventilation without feedback (“blinded”).

**Results & Discussion**

Preliminary results from our first 50 medical students is presented in the table. Using the paired two-tailed t-test, ventilation volume increased from 233.94 ml to 427.94 ml (p < 0.005) after real-time simulation feedback. In addition, percent adequate ventilation (400-700 ml) increased from 20.4% to 60.0% (p < 0.005) after real-time simulation feedback. Ventilations per minute also improved from 9.15 ventilations/minute to 10.5 ventilations/minute, however this was not statistically significant (p=0.383). Students also reported increased confidence in performing BVM ventilation on patients after these simulation sessions.

Our preliminary results show using real-time simulation feedback can objectively improve BVM ventilation performed by 3rd year medical students. Thus, simulation is a useful tool to improve medical student BVM ventilation techniques as they prepare for EPA #12.

**Ethics statement**

The authors declare that all procedures followed were in accordance with the ethical standards of the responsible committee on human experimentation (institutional and national) and with the Helsinki Declaration of 1975 (In its most recently amended version). The IRB determined informed consent was not necessary for participants included in the study.

## A11 Learning and behavior change after a training program based on simulation to enhance bad news delivery

### Gemma Claret Teruel^1^, Jose María Quintillá Martínez^1^, Carmen De la Gala Otero^1^, Carlos Aláez Vasconcellos^1^, David Nadal Miquel^2^, Jaume Pérez Pallarols^1^

#### ^1^ Simulation Program. Sant Joan de Déu Hospital, Barcelona, Spain; ^2^ Social Work Service. Sant Joan de Déu Hospital, Barcelona, Spain

**Correspondence:** Gemma Claret Teruel (gclaret@sjdhospitalbarcelona.org)

**Introduction & Aims**

In 2017 the Patient Experience Team of Sant Joan de Déu Hospital (SJDH) developed a Guide on how to deliver bad news based on professionals’ and families’ contributions. Professionals requested specific training to improve families' experience.

SJDH Simulation Program designed a two-part training program: a 5-hour workshop on relational styles based in Bridge Model and a 8-hour simulation session with professional actors. A debriefing after each scenario allowed participants to share their thoughts and feelings in a safe environment. We promoted deep reflection on different points of view and real difficulties, not the acquisition of a standard technique of communication.

Aims of this study are:

1. Evaluate the experience of the participants during the training program
2. Analyze the perception of learning after the course and over time.
3. Identify behavior changes of the participants in real situations.

**Methods**

Levels 1-3 of Kirkpatrick’s model were used as reference for assessment. Participants filled anonymous surveys at 3 different moments:

- Before the course.
- Immediately after the course: experience (level 1) and learning (level 2).
- 6 months after the course: learning (level 2) and behavior (level 3).

**Results & Discussion**

In 2017 and 2018 we performed 4 editions of the training program with 69 multidisciplinary participants (78% women; 40.3% more than 15 years of clinical experience).

Experience (level 1): The mean of the scores in questions related to this aspect was 4.85 / 5. Learning (level 2): Table 1 shows significant knowledges acquisition during the course and also significant maintenance 6 months later.

Behavior (level 3): The mean of the scores in questions about application of specific behaviors in real situations was 4.1 / 5.

In conclusion, training on how to give bad news based on highly realistic simulation scenarios is an experience well valued by participants, enables the acquisition of new knowledge that is maintained over time and facilitates specific behaviors in real situations.

**Ethics statement**

The authors declare that all procedures followed were in accordance with the ethical standards of the responsible committee on human experimentation (institutional and national) and with the Helsinki Declaration of 1975 (in its most recently amended version). Informed consent was obtained from all patients/participants included in the study.

**Table 1 (abstract A11).** Comparison between the knowledge before the course, immediately after the course and 6 months later

	Learning during the course			Maintenance of the acquired knowledge	
Topic	Punctuation* before (n=62)	Punctuation* after (n=46)	Comparison before/after p	Punctuation* 6 months later (n=20)	Comparison after/6 months later p
I know what aspects I have to prepare before delivering bad news	3	4	<0.001	4	NS
I know what to do after giving the bad news so that families feel accompanied	3	4	<0.001	4	NS
I feel ready to communicate bad news to patients and family	3	4	<0.001	4	NS
I have communication skills to deliver bad news	3	4.14	<0.001	4	NS
I have skills to relate to patients and their families	4	4	0.001	4	0.065
I have confidence in myself to face bad news delivery	4	4.19	<0.001	4	NS

*Median of scores of the participants (between 1 and 5, being 1 in total disagreement and 5 in total agreement)

NS= Non significant

## A12 Maternity VR: preparing expectant parents for labour

### Christopher Gay^1^, Suzanna LAscelles^2^, Janet Cairns^2^, Hayley Rust^2^, Melanie Lee^2^, Melanie Lee^1^, David Wright^1^

#### ^1^Hull Institute of Learning and Simulation, Hull, UK; ^2^Hull and East Yorkshire Hospitals NHS Trust, Hull, UK

**Correspondence:** David Wright (dave.wright@hey.nhs.uk)

**Introduction & Aims**

Childbirth is a unique and varied experience. Current antenatal preparation for parents focuses on the practical aspects of labour and delivery, but does little to address environmental and experiential familiarisation and preparedness. A newly commissioned midwifery-led unit, which valued the contribution of environment, prompted a collaboration with The Hull and East Yorkshire Hospitals (HEY) Maternity Team, to develop a 360 degree virtual reality (VR) experience.

The aim was to better prepare expectant parents and pilot the feasibility of using VR at scale.

**Description**

We used a 360 degree camera (Samsung Gear 360) to capture video of simulated birth experiences, in both standard labour ward and midwifery-led units. This included a general familiarisation and the following key aspects; birthing pool, bed, birthing ball, entonox and inclusion of birth partner. 100 expectant parents viewed the video on VR headsets (Oculus Go), during high throughput, multifunctional antenatal events. This was supported by a printed quick start guide. Data on usability, relevance and effectiveness was collected via questionnaire post event (fig 1).

**Discussion**

Usability was high, with only 4% finding the headset clearly uncomfortable. This is higher than our experience in other settings and may reflect patient demographics i.e. generally young adults who may have less vestibular/ocular comorbidity and greater familiarity with VR the technology. Understanding of professional roles and the processes observed was high at 95% and 97% respectively. 84% report feeling better prepared for their birth experience and global assessment of use was high at 91%. Interestingly, 15% thought a flat screen video would have had the same impact. Our experience suggests that effective 360 degree VR experiences can be delivered to large numbers of participants, instead of physical familiarisation with its inherent constraints. Only 3 headsets were used for this cohort. Success was likely due a number of factors; anticipation of technical pitfalls, using the quick start guide; relevant, time limited content; adequate, trained support staff and a suitable VR platform. We used VR headsets at the lower end of the commercial spectrum, designed for domestic use. We suggest that higher specification technology is not required for this type of experience. The content was designed to be suitable for lower specification devices, utilising a bring your own device (BYOD) smartphone. We intend to pilot this option in future, using other experiences, as a platform for web-based distribution. This may increase engagement and access.

**Ethics statement**

Performed following accepted ethical standards. Formal ethics committee approval not required on discussion with local R and D. Survey data collected with informed consent.

## A13 Non-technical skills, including Leadership and Follower-ship, in a water rescue scenario for the North Wales fire and rescue service

### Toby Jackman, Suman Mitra

#### Cardiff University, Wales, UK

**Correspondence:** Toby Jackman (jackmantw@cardiff.ac.uk)

**Introduction & Aims**

Simulation improves patient safety, individual performance, and team performance, especially in low-frequency, high-acuity scenarios. For the North Wales Fire and Rescue Service (FRS), water rescue is such a scenario: the frequency of water rescue events is low, and yet the acuity and expertise needed at each call is high, with drowning being a common cause of death worldwide. In high-reliability contexts, crew resource management, including aspects of leadership and followership, are critical to success. We aim to develop and run an appropriate, realistic, simulated FRS scenario; to assess overall team non-technical skills; to determine individual participant followership styles and preferred leadership styles; and to assess the extent of correlation of followership styles and preferred leadership styles.

**Methods**

Using accepted frameworks, we designed and delivered a realistic pre-hospital paddle-sport water-rescue team-training in-situ simulation scenario which involved a submerged casualty, immersed casualties, a shoulder dislocation, minor abrasions and lacerations, and a casualty with suspected c-spine injury. A previously validated questionnaire was administered to assess participant non-technical performance, and a second validated questionnaire assessed participant followership styles and preferred leadership styles. FRS member followership styles were categorised into ‘Alienated’, ‘Conformist’, ‘Exemplary’, and ‘Passive’, and preferred leadership styles were categorised into ‘Delegating’, ‘Participating’, ‘Selling’, and ‘Telling’. Participant followership styles and preferred leadership styles were then compared to existing integrated theoretical models.

**Results & Discussion**

All FRS members scored highly in both ‘active engagement’ and ‘independent critical thinking’ domains, which classified all members as ‘exemplary’ followers. Similarly, all FRS members’ preferred leadership styles involved both high relationship behaviour and high task behaviour – a ‘selling’ style. Follower independent critical thinking and leader task behaviour were strongly negatively correlated. This partially supports theoretical integrated models of leadership and followership. This is one of the first attempts to match leadership and followership styles in a real-life healthcare application. Leadership and followership are holonarchical: good leadership is a requirement of good followership, and vice versa. In contexts where other non-technical skills – for example, situational awareness – may be limited by workplace culture, followership interventions may improve team performance.

**Ethics statement**

The authors declare that all procedures followed were in accordance with the ethical standards of the responsible committee on human experimentation (institutional and national) and with the Helsinki Declaration of 1975 (In its most recently amended version). Informed consent was obtained from all participants included in the study.

## A14 Out of hours access to low-fidelity part-task surgical simulators: the trainee perspective

### Andrew Barrie, Andrew Barrie, Alistair Hunter

#### University College Hospital, London, UK

**Correspondence:** Andrew Barrie (andrewbarrie@doctors.org.uk)

**Introduction & Aims**

Low-fidelity part-task computer based surgical simulators, including virtual-reality simulators, are an emerging training tool. They have been shown to provide benefit in the development of surgical techniques and transferable skills for surgical trainees. Despite our hospital providing 0900-1700, Monday-Friday access to a large variety of surgical simulators (laparoscopic, robotic, VR); anecdotally, these were scarcely used. We wanted to explore the factors impeding utilisation.

Hypothesis: Trainees would desire ad-hoc out of hours access to low-moderate fidelity part-task surgical simulators.

**Methods**

A cross-sectional qualitative scoping survey was distributed to all junior doctor staff at a central London university teaching hospital. The self-reporting 26 point online questionnaire was completed by 30 junior doctors (Male:17; F1-2: 10, Trust grade SHO: 7, CST: 1, ST3-4: 5, ST5+: 3, Trust grade SpR: 4) between October 2019 and November 2019. Questions included assessing the importance of out of hours access to trainees and times when trainees would be most likely to use simulators, alongside demographic data.

**Results & Discussion**

The majority (85%) of trainees report that out of hours access to surgical simulators is important to them with 51% suggesting it is extremely important. Given current work scheduling, 85% of trainees suggested they would use surgical simulators between 1700-2000 and there was also demand for 24 hour access (20-44%). Only 1 trainee reported currently using the surgical simulators (once a month) within the available access hours.

The results of this study show that out of hours access to surgical simulators is a highly desirable resource to junior doctors in training. Given current service pressures within the NHS, restricting access times to training doctors proves to be a barrier to the utility of a key training resource. The importance of usable access to simulators cannot be understated with 80% of trainees self-reporting that they do not currently get enough operative experience for their educational needs. Furthermore, 66% of trainees suggested having improved access to surgical simulators would increase the likelihood of joining a surgical speciality highlighting an interesting further research area (factors influencing this). Improving access may aid trainees in making career development decisions and contribute to shaping future specialities.

Although desirable, few trainees (17%) were prepared to pay for out-of-hours access, leaving the onus on centres to devise novel ways of making this sustainable. Our centre is dedicated to exploring this and we endeavour to develop 0800-2000 access.

**Ethics statement**

The authors declare that all procedures followed were in accordance with the ethical standards of the responsible committee on human experimentation (institutional and national) and with the Helsinki Declaration of 1975 (In its most recently amended version). Informed consent was obtained from all patients/participants included in the study. All institutional and national guidelines for the care and use of laboratory animals were followed.

## A15 Place of TrueCPR feedback device in resuscitation learning process. A randomized pilot data

### Lukasz Szarpak^1^, Jacek Smerek^2^, Dominika Dunder^1^, Kacper Kranc^1^, Michal Paprocki^1^, Zuzanna Wiesniewska^1^

#### ^1^Lazarski University, Warszawa, Poland; ^2^Department of Emergency Medical Service, Wroclaw Medical University, Wrocław, Poland

**Correspondence:** Lukasz Szarpak (lukasz.szarpak@gmail.com)

**Introduction & Aims**

The ability to perform high quality chest compressions should be one of the key skills that medical personnel should have. However, according to numerous studies, the depth of chest compressions is in many cases insufficient. The aim of the study was to assess the impact of the use of TrueCPR in the resuscitation teaching process on the quality of chest compressions.

**Methods**

The study was attended by 60 students of the first year of medicine studies. Before starting the study, all participants of the study took part in a training in basic life support. Then they performed a 2-minute cycle of one-rescuer cardiopulmonary resuscitation (Baseline). The participants were then divided into two groups, with the control group being able to practice chest compressions for 10 minutes, while the experimental group performed resuscitation using a TrueCPR feedback device (Physio-Control, Inc., Redmond, WA, USA). One month after the training, participants were asked to re-examine a 2-minute cycle of cardiopulmonary resuscitation (Evaluation phase). Only chest compression parameters were analyzed. The Resusci Anne® QCPR simulator (Laerdal, Stavanger, Norway) was used to simulate the patient requiring resuscitation.

**Results & Discussion**

The chest compression rate achieved the value of 115 vs 126 (p<0.001), adequate chest compression rate (%) was 86 vs 68 (p<0.001), full chest release (%) 96 vs 65 (p<0.001), and correct hand placement (%) 99 vs 99 (p, not significant) in Experimental vs. Control groups, respectively. As for the assessment of the confidence of chest compression quality, 1 month after the training, the evaluation in the experimental group was statistically significantly higher (87 vs 65; p<0.001) than in the control group. Performing chest compressions with a rate above 120 CPM does not affect survival, and may cause faster fatigue of the rescuer, so the most optimal frequency is 100-120 CPM. Perfusion pressure during resuscitation is the result of a difference in chest pressure caused by compression and relaxation of the chest, so it can be assumed that in the experimental group the perfusion pressure would be higher than in the control group, where both the depth of compressions was insufficient and the percentage of complete relaxation of the chest was insufficient. To sum up, the use of CPR feedback devices in the resuscitation teaching process may influence the student's ability to develop correct habits related to chest compressions.

**Ethics statement**

The authors declare that all procedures followed were in accordance with the ethical standards of the responsible committee on human experimentation (Polish Society of Disaster Medicine) and with the Helsinki Declaration of 1975. Informed consent was obtained from all participants included in the study. All institutional and national guidelines for the care and use of laboratory animals were followed.

## A16 Randomised single-blind crossover trial investigating the effectiveness of a new low-cost manikin for central venous catheter insertion

### Katherine Brooks, Daniel Turton, Andrew Birch

#### Barts Health NHS Trust, London, UK

**Correspondence:** Katherine Brooks (katherine.brooks@doctors.org.uk)

**Introduction & Aims**

Central venous catheter (CVC) insertion is an essential procedure. The use of manikins to teach such skills is increasingly common. The CVC manikins available on the market, however, represent a significant cost which may discourage educators from using models for CVC training. We compared a new low-cost manikin [Bartikins], developed in Barts Health NHS Trust, to a well-established CVC manikin [Pharmabotics CVC200].

**Methods**

A randomised single-blind cross-over study testing the hypothesis that the new CVC manikin was significantly more effective than the established manikin at simulating right internal jugular (RIJ) CVC insertion. The primary outcome was “overall effectiveness” as rated by participants expert at CVC insertion. Secondary measures were user-ratings of other aspects of the two manikins. The participants each performed ultrasound-guided (US) RIJ CVC insertion on both the new and the established manikins. The manikin each participant used first was determined using a random number generator. After using a manikin, participants ranked it on a 1-7 scale for overall effectiveness (the primary outcome) together with other aspects of the procedure, with 7 being positive and 1 negative. Results were analysed using Wilcoxon-signed rank test on SPSS [IBM] version 23.

**Results & Discussion**

Primary outcome: our data shows that expert practitioners considered both manikins to be effective overall at simulating CVC insertions. Since both manikins had a median score of 5/7 (p=0.084), we did not demonstrate that either was superior to the other. Secondary outcomes: the manikins scored similarly with small divergences in three areas: the new manikin scored higher for ease of obtaining an ultrasound view (p=0.04) and the established manikin scored higher for simulating tract dilation (p=0.015) and CVC insertion (p=0.024).

These data suggest that experienced practitioners found both manikins effective for simulating CVC insertion and) the new manikin [Bartikins] performs similarly to an established manikin available on the market [Pharmabiotics CVC200]. The new manikin is not yet available to buy but is expected to cost less than 5% of the established manikin used in this research.

**Ethics statement**

The authors declare that all procedures followed were in accordance with the ethical standards of the responsible committee on human experimentation (institutional and national) and with the Helsinki Declaration of 1975 (In its most recently amended version). Informed consent was obtained from all patients/participants included in the study. All institutional and national guidelines for the care and use of laboratory animals were followed.

## A17 Simulation scenario design- A Biggs Dilemma

### Nathan Oliver

#### NHS Lothian, Scotland, UK

**Correspondence:** Nathan Oliver (v1nolive@ed.ac.uk)

**Introduction & Aims**

Peak professional bodies in clinical simulation, such as the International Association for Clinical Simulation and Learning (INACSL) and the Association for Simulated Practice in Healthcare (ASPiH), strongly advocate a robust design process within immersive simulation scenarios in the health care setting. Not only this, but additionally within the process of simulation design and delivery there is an impetus for designers of clinical simulation, emerging both from stakeholders and the professional bodies, to demonstrate the effectiveness of these simulation scenarios and programmes. However to date there has been little research as to the experiences and perceptions of designers themselves as it relates to the factors of scenario design and the measurement of learning. This study sought to understand what methods, if any, simulation scenario designers use to drive and measure learning within immersive, non-assessed simulation scenarios.

**Methods**

Seven experienced clinicians and simulation designers were interviewed across Scotland, Wales, England, and Australia.

The research question was “What methods, if any, do simulation scenario designers use to drive and measure learning within immersive, non-assessed SBE programmes?”

To answer this broad enquiry, several sub questions were asked:

1. What is the perception of the role of ILO’s in the development of simulation scenarios?
2. What methods are used, if any, to ensure ILO’s are fully engaged with within the scenario?
3. How is ILO attainment measured in immersive, training based simulation scenarios?

The researcher professed a interpretivist epistemological position and approached the research with a phenomenologically qualitative design. A series of semi-structured interviews were undertaken with the sample group and the data underwent thematic analysis to draw out emergent themes to respond to the research question.

**Results & Discussion**

The results uncover several different approaches used in designing scenarios and the discussion explored the challenges associated with designing and measuring learning in immersive simulation. There was a clear delineation in scenario designers between those who professed a 'rigid' approach and other who advocated a much more 'fluid' approach in the context of both formulating immersive simulation scenarios and measuring the learning that occurred.

The paper concludes by making several key recommendations in the development of simulation scenario design.

Firstly in embracing the term ‘intended learning outcome’ as a unifying term to encompass two conflicting approaches in the delivery of simulation. Secondly in promoting broader stakeholder engagement in designing goals of interventions in workplace based education as a kind of proxy to measuring deeper learning processes.

**Ethics statement**

The authors declare that all procedures followed were in accordance with the ethical standards of the responsible committee on human experimentation (institutional and national) and with the Helsinki Declaration of 1975 (In its most recently amended version). Informed consent was obtained from all patients/participants included in the study. All institutional and national guidelines for the care and use of laboratory animals were followed.

## A18 Something for everyone: what do participants from three different professional backgrounds take away from the same simulated ward round?

### Jennie Higgs, Alison Mackie, Chris O'Shea, Chris Schnieke-Kind

#### NHS Lothian, Scotland, UK

**Correspondence:** Jennie Higgs (jenniehiggs@nhs.net)

**Introduction & Aims**

Doctors, nurses and pharmacists must immediately integrate into the multidisciplinary team (MDT) when they commence their working lives. Ward round competence is a vital skill across these disciplines. Despite being exposed to numerous ward rounds during training, local data suggests that final year medical and nursing students, along with pre-registration pharmacists, feel under prepared for their future role upon graduation or registration.

In NHS Lothian we have created a collaborative, high fidelity, simulated ward round for these groups. Participants act up into their prospective roles and rehearse ward round skills in a safe learning environment. We were interested to know what participants took from this experience and whether there was inter-professional variation.

**Description**

Participants were asked to reflect on the simulated ward round, and their wider experiences, via a structured debrief. The ward round deliberately challenged participants by introducing distractions and unexpected events. The debrief aimed to emphasise the importance of managing tasks, working as a team and communicating within that team to all participants.

At the end of the session participants were asked to individually document up to six Take Home Messages (THMs) from the ward round. We were interested in what these were, whether they were aligned to our learning objectives and whether differences existed between professions.

Thematic analysis separated the data into eight themes, with several sub-themes: Communication, Task Management, Team, Knowledge, Preparedness, Patient, Accessing help, and Documentation (Table 1). The proportion of THMs coded to each theme was different between professions with the three largest proportions for each profession being:

- Medical students: Task Management (35.5%), Team (26.0%), Preparedness (17.1%)
- Nurses: Communication (48.3%), Task Management (25.5%), Knowledge (13.4%)
- Pharmacists: Communication (86.8%), Task Management (5.3%), Team (5.3%).

**Discussion**

Participants’ THMs aligned closely to our learning objectives. All three professions reported similar but not identical learning. Medical students mentioned Task Management most frequently. This was unsurprising as the ward round is a key opportunity to agree with their consultant a management plan for each patient. As expected, nurses and pharmacists included communication most frequently indicating that the ward round provides a key opportunity to communicate specific care issues with the MDT.

The simulated ward round is rated highly by participants. Our analysis demonstrates that three separate professions can take away clear learning points which will contribute to their ability to transition into their prospective roles. We are proud to be able to offer a truly inter-disciplinary learning experience.

**Ethics statement**

The authors declare that all procedures followed were in accordance with the ethical standards of the responsible committee on human experimentation (institutional and national) and with the Helsinki Declaration of 1975 (In its most recently amended version). Informed consent was obtained from all patients/participants included in the study. All institutional and national guidelines for the care and use of laboratory animals were followed.

## A19 Stories of Success: Demonstrating improved performance in junior doctors after simulation

### Heather Stirling, Nathan Oliver

#### NHS Lothian, Scotland, UK

**Correspondence:** Heather Stirling (heather.stirling1@nhs.net)

**Introduction & Aims**

A large amount of the credibility of immersive simulation rests on the assumption that the experience of simulation positively impacts on clinical behaviour. Whilst the literature has demonstrated an impact on confidence in participants undergoing simulation, there is a distinct lack of evidence which demonstrates direct clinical impact in these cohorts. With significant amounts of resources required to run simulation programmes internationally, demonstrating that this positively impacts on junior doctors' clinical performance is essential.

In 2013, a simulation programme was set up for first year junior doctors, to respond to areas of the curriculum which they were finding hard to evidence. This programme consists of three sessions that run over the year, focusing on technical and non-technical elements of patient care.

This study sought to look for what impact, if any, did the simulations programme have on the clinical performance of first year junior doctors in NHS Lothian.

**Methods**

This was a qualitative study utilising a narrative enquiry approach. Between 2013 and 2018, a voluntary questionnaire was sent to all junior doctors at completion of their first year of training. This questionnaire asked them to briefly describe, if possible, two specific accounts of when they were able to apply what they had learned during their simulation experience into their clinical setting. Questionnaires were coded and analysed using thematic analysis.

**Results & Discussion**

264 junior doctors (n=264) returned their completed questionnaire giving a total of 528 narrative accounts for analysis. The narratives draw strong, contextualised links from what was learned in simulation to direct positive impacts on their performance. The following five themes were identified: situational awareness, teamwork, communication, legal decision making, and the acute management of the unwell patient. The most commonly cited themes were impacts in performance in acute management (458 separate accounts), communication (374 accounts), and teamwork (364 accounts). The accounts in this study express a clear link between their experience in a simulation programme and their direct clinical performance, often months after the learning experience. Whilst acknowledging that the responses received in this study are inherently subjective in nature, it is asserted that the large sample size, along with the 5 year span of data collection, add a level of validity in responding to the question at the centre of this enquiry. Simulation has been seen to positively contribute directly to increased clinical performance, leading to the logical extension of impacting where it truly matters - safer patient care.

**Ethics statement**

The authors declare that all procedures followed were in accordance with the ethical standards of the responsible committee on human experimentation (institutional and national) and with the Helsinki Declaration of 1975 (In its most recently amended version ). Informed consent was obtained from all patients/participants included in the study.

## A20 Using simulation to improve paediatric trainees’ confidence in performing common neonatal procedures

### Sarah Williamson, Matthew Nash

#### Birmingham Women's and Children's NHS Foundation Trust, Birmingham, UK

**Correspondence:** Sarah Williamson (sarahlouise.williamson@nhs.net)

**Introduction & Aims**

Undertaking your first neonatal job as a new paediatric trainee can be a daunting experience. Learning to perform common neonatal procedures is an essential part of neonatal care. Neonates are some of the smallest and most vulnerable patients. It is therefore essential these procedures are performed competently and in accordance with national guidance to reduce the risk of complications, for example catheter associated infection.

We therefore designed a neonatal clinical skills simulation course for first year paediatric trainees, aiming to improve trainee’s confidence in undertaking commonly performed neonatal procedures, whilst simultaneously improving patient safety.

**Description**

We designed and implemented a 1 day neonatal clinical skills simulation course for first year paediatric trainees at the start of their first neonatal placement. This consisted of 4 small group simulation teaching sessions, giving trainees the opportunity to learn and practice common neonatal procedures in a realistic environment.

Skills covered included;

1. Airway management and intubation
2. Central venous and arterial access
3. Chest drain insertion and needle thoracentesis
4. Capillary blood sampling, intraosseous needle insertion and peripheral arterial access

These practical skills were supported by small group discussions on indications, insertion guidelines and infection control considerations. In addition we delivered small group teaching sessions on stabilisation of the extreme preterm infant in the first hour of life and cerebral function monitoring (CFM).

**Discussion**

9 first year paediatric trainees attended the pilot course in September 2018. We asked trainees to rate their confidence before and after the course on a scale of 1 to 5, 5 being very confident. All trainees reporting feeling more confident in all skills after attending the course (Figure 1). Most notably, trainee’s confidence improved from an average of 2.5 to 4.3 after attending the airway management and intubation simulation session. 100% of trainee’s rated the simulation course as useful or very useful (4-5/5). Feedback comments included ‘enjoyed having focused time to learn and practise clinical skills’, ‘I feel a lot more confident now’ and ‘very helpful and informative, loved it!’ 100% of trainee’s stated they would recommend the course to their colleagues.

Giving trainees the opportunity to learn and practice these skills in a safe, simulated environment improved their knowledge and confidence, theoretically leading to improved patient safety. Due to the success of the pilot simulation course, it is now ran twice yearly as part of the deanery wide Speciality Training year 1 (ST1) teaching programme.

**Ethics statement**

The authors declare that they have followed the guidelines for scientific integrity and professional ethics. The article does not contain any studies with human or animal subjects.

**Figure 1 (abstract A20).**
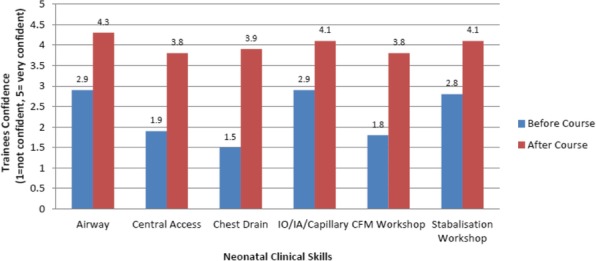
Trainees Confidence

## A21 'You’re going to be a doctor and you can’t tell right from left!' A phenomenological analysis of medical students (in)abilities in distinguishing right from left

### Gerard Gormley, Carl Brennan, Martin Dempster

#### Queen's University Belfast, Belfast, Northern Ireland

**Correspondence:** Gerard Gormley (g.gormley@qub.ac.uk)

**Introduction & Aims**

Wrong-sided procedures represent some of the most catastrophic errors in healthcare such as removal of the wrong kidney. Though multifaceted in origin, human error is considered an important root cause. Evidence indicates that a significant proportion of our population, including medical students, experience difficulty with left/right discrimination (LRD). Given that not all medical students have equal LRD ability, there have been calls to raise its awareness in medical education. It remains unknown what the experiences of medical students when conducting LRD tasks. Elucidating such experiences may provide new understanding to these challenges and guide future pedagogical practice. The aim of this study was to gain deep insights into medical students lived experiences of LRD.

**Methods**

In order to bring to the surface individuals LRD experiences, hermeneutic phenomenology was deemed conceptually a good fit. Using a purposeful sampling method of 10 (as typical in phenomenological-based studies) medical students, with various LRD abilities, were invited to participate. Interviews were transcribed and analysed using the Template Analysis approach to generate research themes. The research team were continually reflexive.

**Results & Discussion**

Analysis yielded four main themes 1) Discriminating right from left: An unconscious or conscious task? 2) ‘What...you can’t tell right from left?’: an undesirable skill deficit 3) Concealment 4) ‘But you’re going to be a doctor!’ Impact on professional identify formation.

For many LRD is an unconscious effortless process. However, for a significant number of medical students it represents a relatively challenging task. Individuals who experienced difficulty with LRD felt ‘different’, often embarrassed and stigmatised. They imagined that their ‘skill deficit’ would increase their proneness in making laterality errors. Such circumstances triggered a critical reflection on their ability on being a competent doctor and questioned their suitability for future career specialities (e.g. surgery).

For the first time in the literature, this study provides a nuanced understanding of how individuals discriminate right from left. Individuals who are challenged with LRD, have to carry out a complex conscious process in order to determine right from left. Medical education needs to respond by raising the awareness of this challenge that many medical students face. Even in apparent low-level risk situations, such individuals need to be provided with techniques such as tactical pauses and seeking cross-checks to ensure that they have made the correct laterality decision. Such techniques could be introduced into simulation teaching that emphasises human factors training. It’s not ‘right’ to be ‘left’ in ignorance about LRD.

**Ethics statement**

The authors declare that they have followed the guidelines for scientific integrity and professional ethics. The article does not contain any studies with human or animal subjects. Informed consent was obtained from all patients/participants included in the study. All institutional and national guidelines for the care and use of laboratory animals were followed.

